# Supplementary material for: HSFs drive transcription of distinct genes and enhancers during oxidative stress and heat shock
Source: Nucleic Acids Res. 2022 Jun 10;50(11):6102–15. doi: 10.1093/nar/gkac493 (PMC9226494; doi:10.1093/nar/gkac493)
Supplement: gkac493_Supplemental_File [file gkac493_supplemental_file.pdf]

Supplementary Materials for

HSFs drive transcription of distinct genes and enhancers during oxidative stress and heat shock

Samu V Himanen[1,2], Mikael C Puustinen[1,2], Alejandro J Da Silva[1,2], Anniina Vihervaara[3], Lea Sistonen[1,2*]

Correspondence to: lea.sistonen@abo.fi

This PDF file includes:

Figs. S1 to S9

Table S1

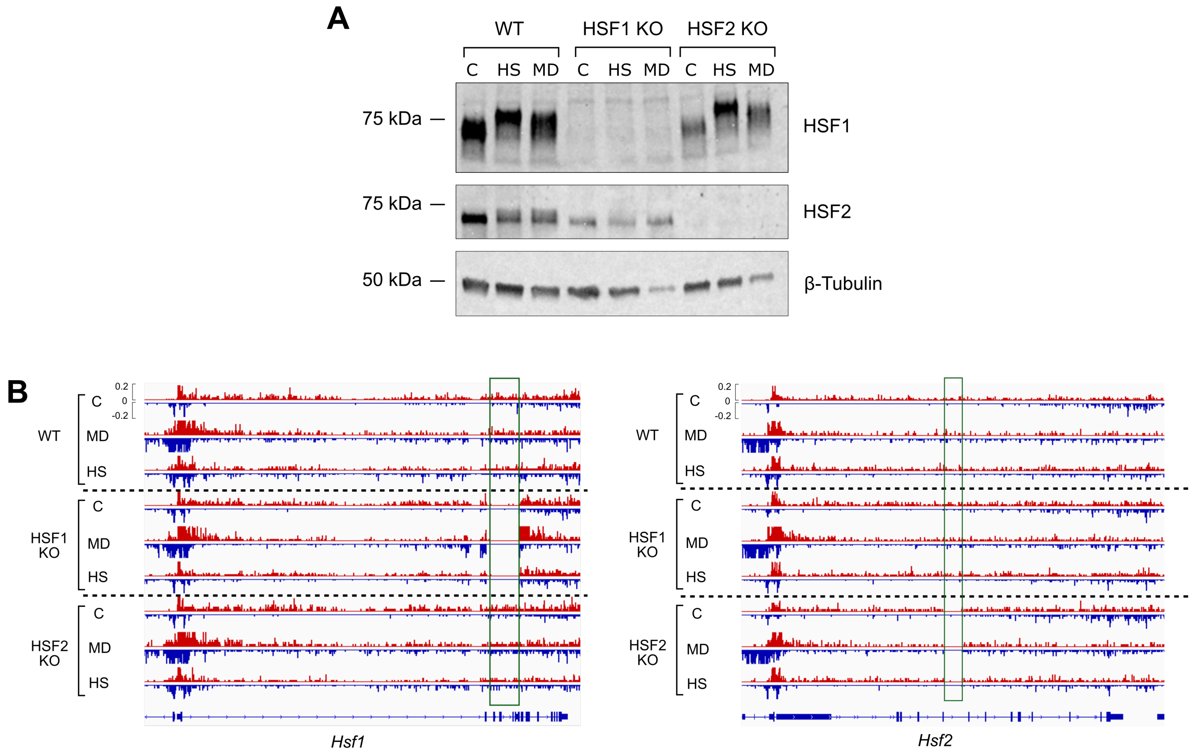

Figure S1. Validation of HSF1 and HSF2 knock out MEFs. (**A**) Western blot was used to determine the levels of HSF1 and HSF2 in wild-type (WT), HSF1 knock-out (HSF1 KO) and HSF2 knock-out (HSF2 KO) MEFs that were exposed to menadione (MD, 30 μM, 2 h) or to heat shock (HS, 42°C, 1 h). β-tubulin was used as a loading control. (**B**) PRO-seq profiles of *Hsf1* and *Hsf2* genes in WT, HSF1 KO and HSF2 KO MEFs that were exposed to menadione or heat shock. Green rectangles indicate regions of HSF1 and HSF2 genes that were deleted to create KO mice, from which the cell lines used in this study have been derived. C: control.

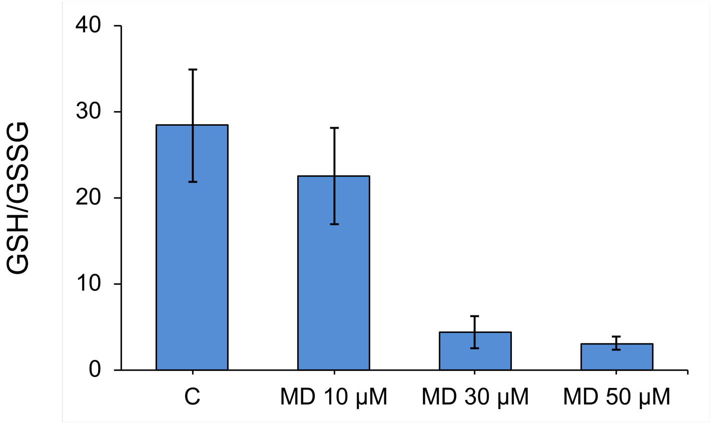

Figure S2. Measurement of GSH/GSSG ratio in menadione-treated cells. MEFs were treated with different concentration of menadione for 2 h to induce oxidative stress. Next, the level of oxidative stress was assessed by measuring the ratio between reduced and oxidized glutathione (GSH/GSSG).

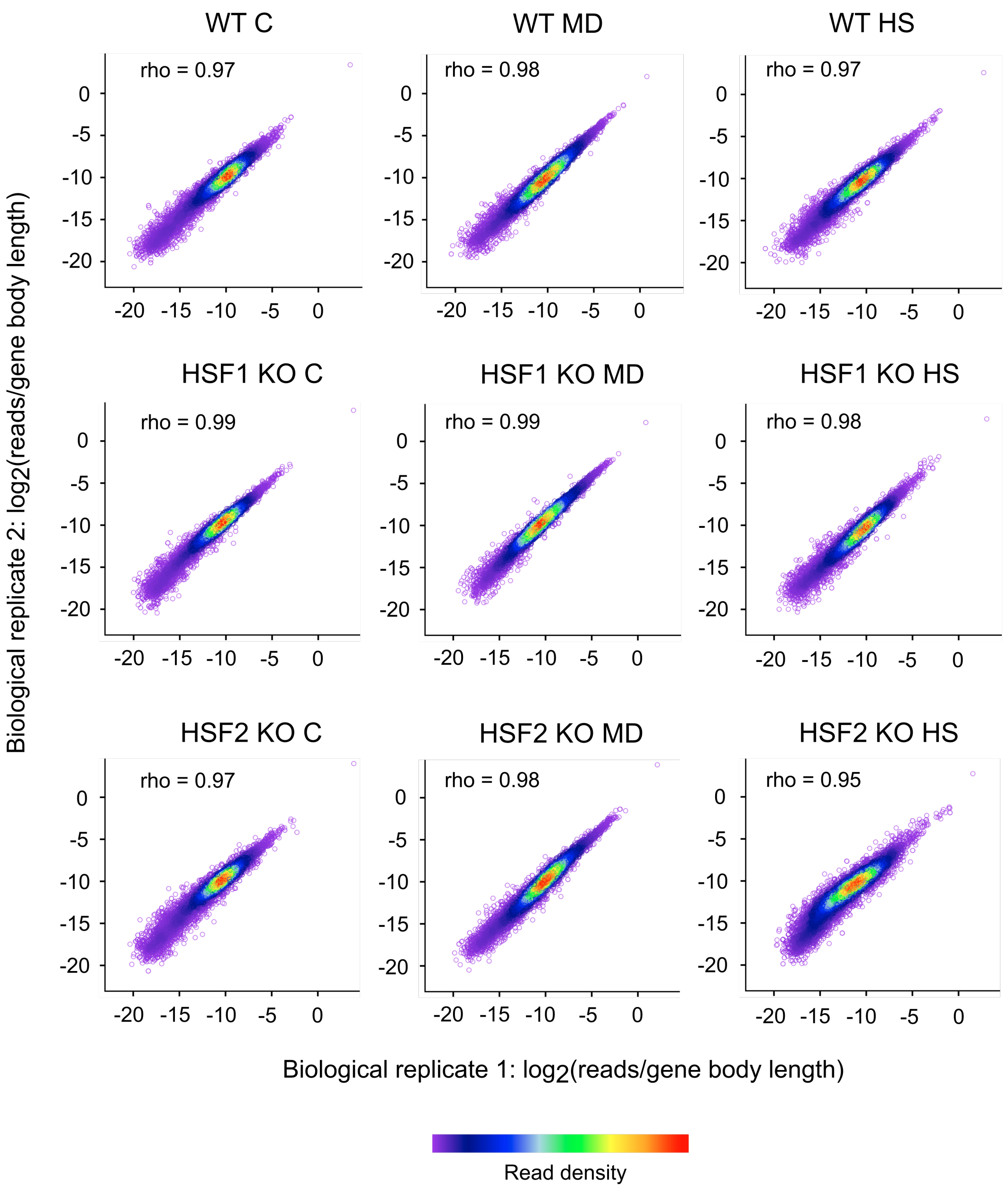

Figure S3. Correlation between biological PRO-seq replicates. PRO-seq was performed in wild-type (WT), HSF1 knock-out (HSF1 KO) and HSF2 knock-out (HSF2 KO) MEFs that were exposed to oxidative stress induced by menadione (MD, 30 µM, 2 h) or to heat shock (HS, 42°C, 1 h). Correlation plots of gene body transcription are shown between two biological PRO-seq replicates. Rho indicates Spearman rank correlation.

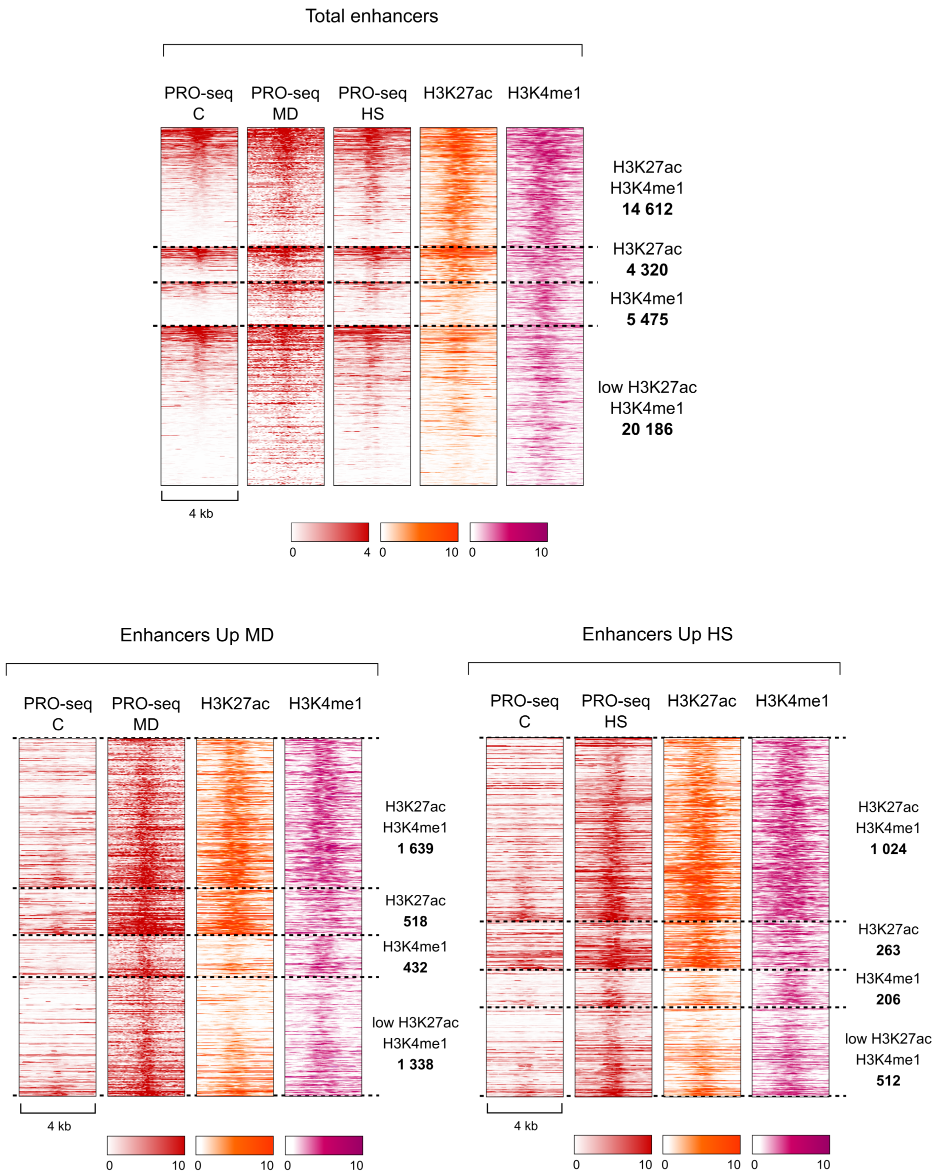

Figure S4. Enhancers detected by PRO-seq contain enhancer-associated histone marks, H3K27ac and H3K4me1. PRO-seq was used to identify transcriptionally active enhancers in MEFs treated with menadione (MD, 30 µM, 2 h) or heat shock (HS, 42°C, 1 h). Enhancers were analyzed for their content of histone marks H3K27ac and H3K4me1, both of which are known to be enriched in enhancers (42, 43). Histone marks were analyzed separately from total enhancers and upregulated enhancers. Intensity of the signal in the heatmaps indicates the number of tags per bin. Bin size was set to 50 bp. C: control.

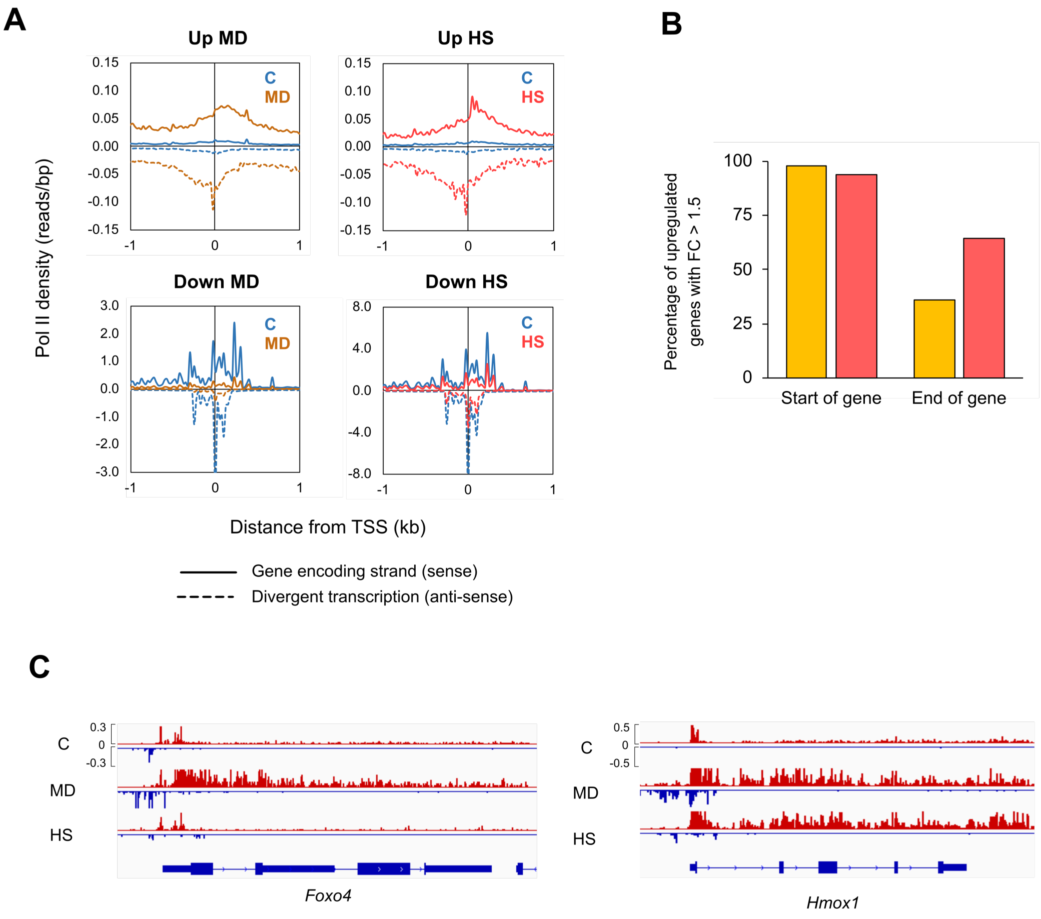

Figure S5. Profiles of Pol II are similar in enhancers but different in genes between oxidative stress- and heat-treated cells. (**A**) PRO-seq was performed in MEFs that were exposed to oxidative stress induced by menadione (MD, 30 µM, 2 h) or to heat shock (HS, 42°C, 1 h). Average density of Pol II was analyzed within enhancers that were upregulated or downregulated by menadione or heat shock. Pol II density was measured separately for plus (solid line) and minus (dotted line) strands. (**B**) Fold changes (FC) of upregulated genes in menadione- and heat-treated cells were determined in start and end of the genes. After this, the percentage of genes that displayed FC over 1.5 in gene start or end were calculated. Start of the gene was defined as a 2 kb window starting 0.5 kb downstream from TSS. End of the gene was defined as a 2kb window upstream of CPS. (**C**) PRO-seq profiles of *Foxo4* and *Hmox1* genes in cells exposed to menadione and heat shock. C: control, *Foxo4*: fork head box O4, *Hmox1*: and heme oxygenase 1.

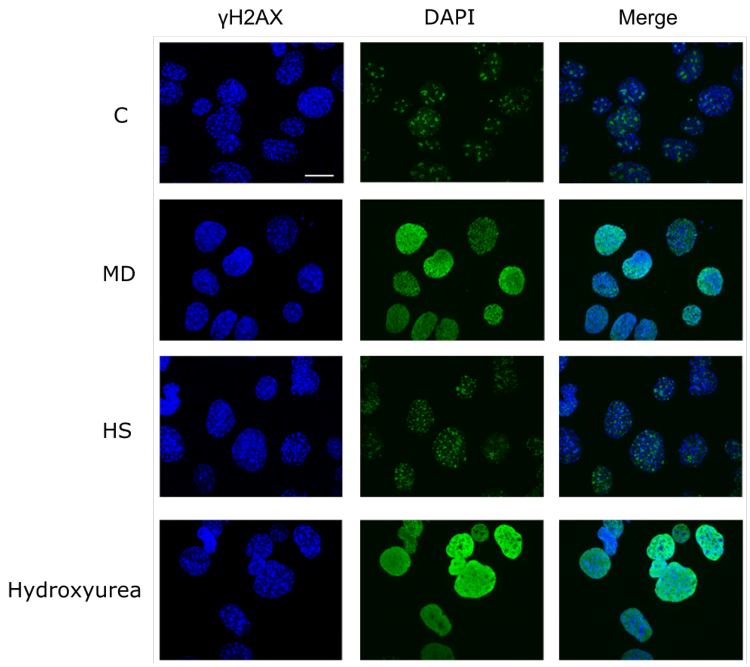

Figure S6. Oxidative stress induced by menadione causes DNA damage. MEFs were exposed to oxidative stress induced by menadione (MD, 30 μM, 2 h) or to heat shock (HS, 42°C, 1 h). The amount of DNA damage was determined by immunofluorescence staining of the phosphorylated H2AX (γH2AX). Hydroxyurea (2 mM, 17 h) was used as a positive control to induce DNA damage. DAPI was used to stain DNA. All images correspond to maximum intensity projections. Scalebar: 20 μm. C: control.

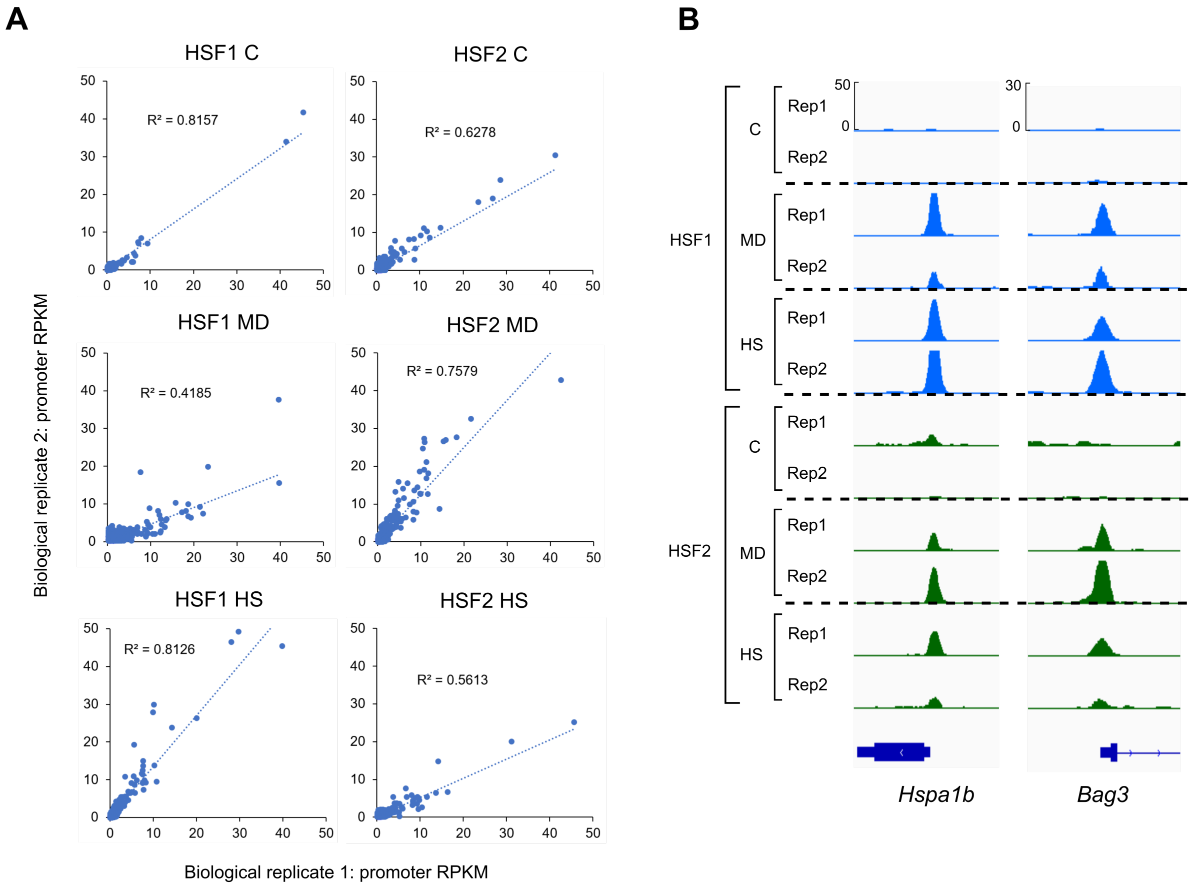

Figure S7. Correlation between biological ChIP-seq replicates. Antibodies against HSF1 and HSF2 were used to perform ChIP-seq in MEFs that were exposed to menadione (MD, 30 μM, 2 h) or to heat shock (HS, 42°C, 1 h). (**A**) Correlation plots of RPKM values at promoters are shown between two biological ChIP-seq replicates. Promoters were defined as 0.5 kb windows centered around TSSs. (**B**) HSF1 and HSF2 occupancies at the *Hspa1b* and *Bag3* promoters are shown in two biological replicates. HSF1 and HSF2 signals in the profiles were normalized to total read counts.

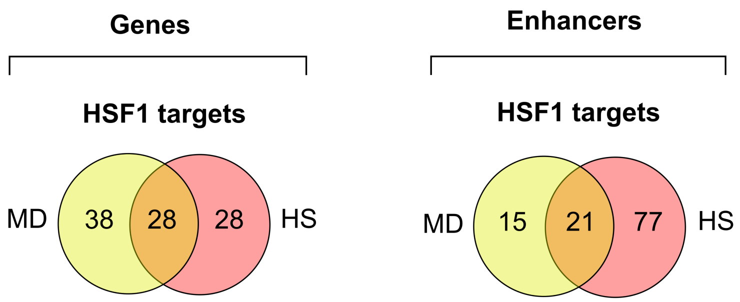

Figure S8. HSF1 regulates stress-specific sets of genes and enhancers. Comparison between menadione- and heat-inducible targets of HSF1 revealed genes and enhancers that are regulated by HSF1 in a stress type-specific manner.

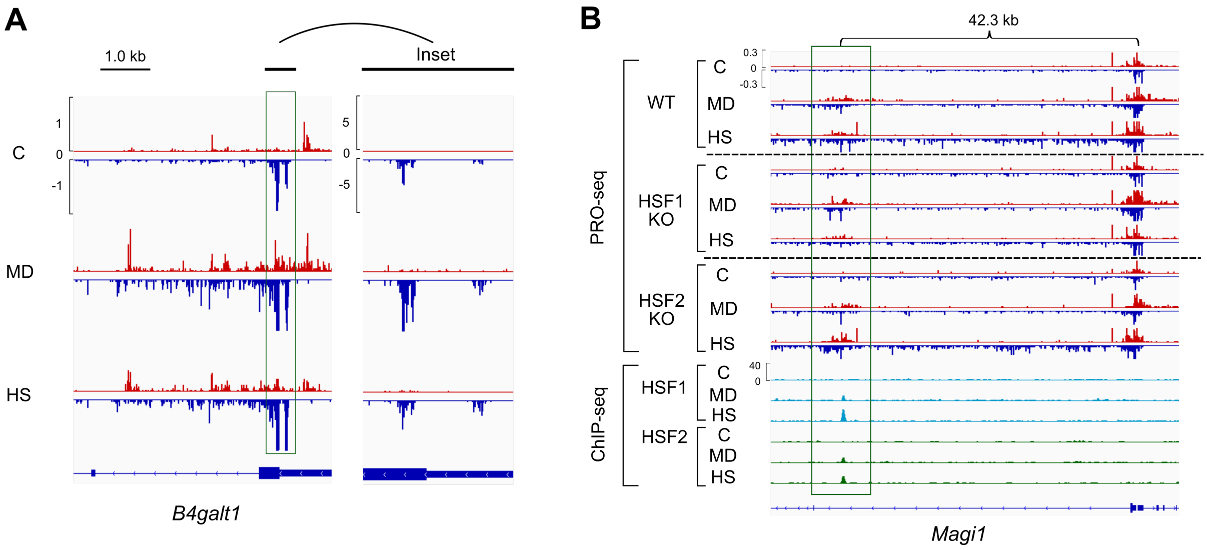

Figure S9. Direct target enhancer of HSF1 is located in the vicinity of the HSF1-dependent gene, *Magi1*. (**A**) PRO-seq profile of *B4galt1* gene showing two distinct TSSs. (**B**) PRO-seq and ChIP-seq profiles of *Magi1* gene and its downstream enhancer. Enhancer is framed with green rectangle. Enhancer is regulated by direct binding of HSF1, while *Magi1* gene is devoid of promoter-bound HSF1. C: control; MD: menadione, 30 μM, 2 h; HS: heat shock, 42°C, 1 h; *B4galt*1: beta-1,4-galactosyltransferase 1; *Magi1*: membrane associated guanylate kinase, WW and PDZ domain containing 1.

Table S1. List of oxidative stress- and heat shock-inducible target genes of HSF1 and HSF2. Combination of PRO-seq and ChIP-seq was used to identify genes in MEFs that are regulated through direct binding of HSF1 and HSF2 during oxidative stress induced by menadione (MD, 30 μM, 2 h) or heat shock (HS, 42°C, 1 h). Genes in the list are ranked in the descending order according to their fold change (FC) in wild-type MEFs.

HSF1 target genes in HS		HSF1 target genes in MD		HSF2 target genes in HS		HSF2 target genes in MD	
Gene name	FC in WT	Gene name	FC in WT	Gene name	FC in WT	Gene name	FC in WT
Hspa1a	308.8	Hspa1l	47.7	Adgra3	2.9	Bst2	3.7
Hspb1	266.3	Mest	37.1	Slc35e2	2.1	Txnip	2.0
Hspa1b	112.0	Lrrc61	7.6	Ints2	2.0	Mcoln1	1.9
Hsph1	76.4	Hsph1	6.8	Xpnpep3	1.8	Cnpy4	1.9
Dnaja4	48.9	Hikeshi	5.3	Abcc5	1.8	Commd3	1.8
Hspa1l	23.7	Rbm42	5.2	Ptges3	1.7	Ube2g2	1.8
Dnajb1	20.7	Mns1	4.9	Vipas39	1.6	Aox1	1.8
Hsp90aa1	18.9	Slc25a38	4.9			Aptx	1.8
Hspe1	18.3	Nfkbid	4.7			Dnajb5	1.7
Hspa4l	14.8	Bst2	3.7			Atg3	1.6
Bag3	13.5	Gm10069	3.7			Hsp90b1	1.5
Mns1	13.0	Zscan29	3.7				
Dnaja1	9.2	Gm13830	3.6				
Swt1	8.9	Kctd18	3.4				
Hspd1	6.6	Saraf	3.4				
Serpinh1	6.3	Gm6297	3.3				
Gm10069	6.3	Rras	3.2				
Hspb8	5.7	Cacybp	3.2				
Stip1	5.6	Gcnt2	3.0				
Uspl1	4.6	Tmem33	2.9				
Hspa8	4.5	B4galt2	2.9				
Lman2l	3.9	Arl6ip4	2.7				
Kctd18	3.9	Plin2	2.7				
Ahsa1	3.9	Hspa4l	2.6				
P4ha1	3.8	Hsp90ab1	2.5				
Hsp90ab1	3.8	Nr1h2	2.5				
Hikeshi	3.8	Ube2b	2.4				
Cacybp	3.7	Stip1	2.4				
Fkbp4	3.7	Hspd1	2.3				
Chordc1	3.6	Chordc1	2.3				
Mrfap1	3.6	Abcc5	2.2				
St13	3.4	Dbndd2	2.2				
Tmem33	3.4	Rnf34	2.2				
Ubqln1	3.2	Atp6v1a	2.2				
Adgra3	2.9	Actr5	2.2				
Trmt1l	2.8	Cct7	2.1				
Ube2g2	2.6	Slc35e2	2.0				
Chchd2	2.6	Chrac1	2.0				
Gm6297	2.6	Fkbp4	2.0				
Azi2	2.4	Ppid	2.0				
Slc35e2	2.1	Mcoln1	1.9				
Pradc1	2.1	Cnpy4	1.9				
Snx3	2.1	Swt1	1.9				
Rab32	2.0	Serpinh1	1.9				
Ints2	2.0	Azi2	1.9				
Lrrc61	2.0	P4ha1	1.8				
Particl	2.0	Zfp46	1.8				
Serpinb6a	1.9	4833417C18Rik	1.8				
Xpnpep3	1.8	Vipas39	1.8				
Abcc5	1.8	Ube2g2	1.8				
Fbxl14	1.8	Aox1	1.8				
Trim65	1.7	Aptx	1.8				
Prss23	1.7	Esco2	1.7				
Vipas39	1.6	Ddx59	1.7				
Ubb	1.6	Dnajb5	1.7				
Dennd1b	1.6	Rpa2	1.6				
		Sars	1.6				
		Ints2	1.6				
		Serpinb6a	1.6				
		Tcp1	1.6				
		Ppwd1	1.6				
		Xpnpep3	1.6				
		Snx3	1.5				
		Atp5j	1.5				
		Sec62	1.5				
		Hsp90b1	1.5				
